# Altered expression of the Cdk5 activator-like protein, Cdk5α, causes neurodegeneration, in part by accelerating the rate of aging

**DOI:** 10.1242/dmm.031161

**Published:** 2018-03-01

**Authors:** Joshua Spurrier, Arvind Kumar Shukla, Kristina McLinden, Kory Johnson, Edward Giniger

**Affiliations:** 1 National Institute of Neurological Disorders and Stroke, National Institutes of Health, Bethesda, MD 02892, USA; 2 The Johns Hopkins University/National Institutes of Health Graduate Partnership Program, National Institutes of Health, Bethesda, MD 02892, USA

**Keywords:** Neurodegeneration, Aging, Cdk5, *Drosophila*, p35, Cdk5α

## Abstract

Aging is the greatest risk factor for neurodegeneration, but the connection between the two processes remains opaque. This is in part for want of a rigorous way to define physiological age, as opposed to chronological age. Here, we develop a comprehensive metric for physiological age in *Drosophila*, based on genome-wide expression profiling. We applied this metric to a model of adult-onset neurodegeneration, increased or decreased expression of the activating subunit of the Cdk5 protein kinase, encoded by the gene *Cdk5α*, the ortholog of mammalian p35. *Cdk5α*-mediated degeneration was associated with a 27-150% acceleration of the intrinsic rate of aging, depending on the tissue and genetic manipulation. Gene ontology analysis and direct experimental tests revealed that affected age-associated processes included numerous core phenotypes of neurodegeneration, including enhanced oxidative stress and impaired proteostasis. Taken together, our results suggest that *Cdk5α*-mediated neurodegeneration results from accelerated aging, in combination with cell-autonomous neuronal insults. These data fundamentally recast our picture of the relationship between neurodegeneration and its most prominent risk factor, natural aging.

## Abstract (precis)

**Editor's choice:** By developing a comprehensive and quantitative metric for physiological age of *Drosophila*, we show that a neurodegeneration mutant produces its effects in part by accelerating the absolute rate of aging.

## INTRODUCTION

Age is the most prominent risk factor for neurodegenerative disease (NDD), but the relationship between neurodegeneration and aging is complex and controversial. Although each NDD has its own unique hallmarks, most degenerative diseases share a variety of cellular phenotypes with aging, including disrupted proteostasis, impaired cellular trafficking and increased sensitivity to oxidative stress. NDD, however, is not merely brain aging; not every old individual develops NDD. A productive understanding of the pathogenesis of NDD will require that we untangle the relationship between degeneration and aging.

Cyclin-dependent kinase 5 (Cdk5) has been linked to multiple NDDs, including Alzheimer's disease (AD), amyotrophic lateral sclerosis (ALS) and Parkinson's disease (PD) (Nguyen et al., 2001; Patrick et al., 1999; Qu et al., 2007). Cdk5 is an atypical CDK that has no role in cell cycle progression, but rather acts exclusively in postmitotic cells. Cdk5 does not depend on a canonical cyclin for activation; instead, its kinase activity requires binding to an activator that has a three-dimensional fold similar to cyclins, but little sequence similarity. In mammals, there are two major Cdk5 activators, p35 and p39, whereas *Drosophila* has only a single activator, encoded by the gene *Cdk5α*, that is closely related to mammalian p35 and p39, and is sometimes referred to as ‘D-p35’ (Connell-Crowley et al., 2000; Tsai et al., 1994). In the absence of *Cdk5α*, Cdk5 kinase is expected to be functionally silent in *Drosophila* (Connell-Crowley et al., 2000) and, in all cases tested to date, the mutant phenotypes of *Drosophila Cdk5* and *Cdk5α* have been indistinguishable (Connell-Crowley et al., 2007; Kang et al., 2012; Kissler et al., 2009; Nandi et al., 2017). Expression of *p35* and *p39* in mammals, and of *Cdk5α* in *Drosophila*, is largely restricted to neurons, localizing Cdk5 activity to the nervous system (Connell-Crowley et al., 2000; Tang et al., 1995; Tsai et al., 1994). In addition to spatial regulation by activator availability, the activity of Cdk5 is further regulated by the phosphorylation status of the kinase and its activator subunits. Upon binding to Cdk5α, Cdk5 autophosphorylates its activator, targeting it for proteasomal degradation. Cdk5 also inhibits the activity of other kinases that share a similar target sequence preference (‘proline-directed’ protein kinases), such that loss of Cdk5 activity can lead indirectly to enhanced phosphorylation of some Cdk5-target sites due to failure to suppress activity of those other kinases (Anderton et al., 2001; Morfini et al., 2004; Zheng et al., 2007; also, see Discussion for more detailed consideration of this phenomenon). Proper regulation of Cdk5 activity is essential to maintaining normal neuronal function and homeostasis (McLinden, 2012).

Deregulation of Cdk5 activity contributes to a variety of NDDs. Altered Cdk5 activity leads to hyperphosphorylation of tau, inducing formation of neurofibrillary tangles associated with AD (Patrick et al., 1999), and of neurofilament, as in ALS (Nguyen et al., 2001). Altering Cdk5 activity also hyperphosphorylates and inhibits peroxiredoxin 2 (Prx2), an antioxidant enzyme; elevated levels of phosphorylated Prx2 were found in brain tissue of PD patients (Qu et al., 2007). Consistent with its links to multiple NDDs, both gain and loss of Cdk5 function cause neuronal death in culture, and cause neurodegeneration in mouse models (Cruz et al., 2003; Qu et al., 2007; Takahashi et al., 2010; Zheng et al., 2007). Similarly, we have shown previously that *Cdk5α* loss of function in the fly produces degeneration-like phenotypes that mimic cellular phenotypes observed in human disease (Trunova and Giniger, 2012). This is consistent with a large and growing literature documenting that the fly is a valuable model for dissecting the molecular mechanisms underlying the cascade of events in human NDDs (Feany and Bender, 2000; Iijima et al., 2004; Watson et al., 2008). While investigating *Cdk5α*-associated neurodegeneration in the fly, we noted that many of its central phenotypes resemble early onset of normal aging phenotypes (Trunova and Giniger, 2012). As in human disease, this underscored the need to distinguish degeneration from aging, both analytically and mechanistically.

Here, we investigate the effect of increasing, or decreasing, the expression of *Cdk5α* in *Drosophila*. This is expected to have the effect of increasing or decreasing Cdk5 kinase activity, respectively (Connell-Crowley et al., 2000). We first demonstrate that either gain or loss of *Cdk5α* causes death of neurons in a learning and memory center of the *Drosophila* brain [the mushroom body (MB)], and is associated with characteristic phenotypes of degeneration and aging, including impaired autophagy, progressive loss of motor function and shortening of lifespan. We then used gene expression profiling to develop an unbiased and quantitative metric for physiological age. Applying this metric reveals that altering the level of *Cdk5α* accelerates the intrinsic rate of aging of the fly. Last, we further tested this hypothesis and show that an age-dependent phenotype identified by the expression profiling – sensitivity to oxidative stress – also shows aging-like changes in flies with altered *Cdk5α* levels. Taken together, our data suggest that neurodegeneration in response to altered expression of *Cdk5α* arises from a combination of the direct effects of accelerated aging in concert with non-aging pathologies, induced by the altered pattern of cytoplasmic protein phosphorylation.

## RESULTS

### Loss or gain of Cdk5α causes accelerated neuron loss with age

**Fig. 1.**
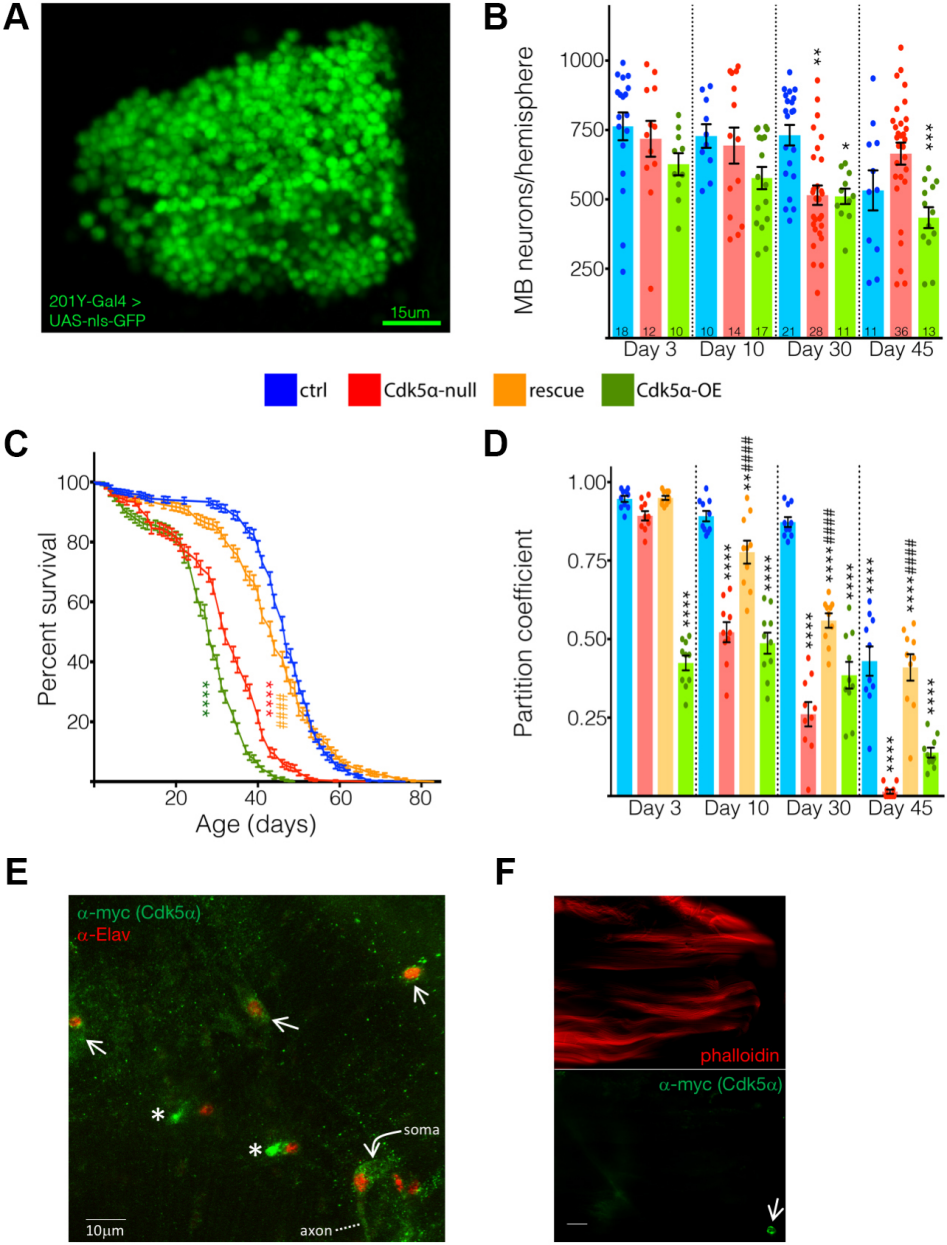
**Gain and loss of Cdk5α induces overt neurodegeneration and degenerative phenotypes.** (A) Projected confocal image of MB neuron nuclei labeled with nls-GFP. (B) Altered Cdk5α levels lead to progressive loss of MB neurons. The number of *201Y>nls-GFP*-positive MB neurons per hemisphere is presented as means±s.e.m., along with individual counts. For each genotype and time point, the number of hemispheres analyzed is presented at the bottom of the bar. Significant differences are relative to the day 3 control. (C) Altered expression level of *Cdk5α* leads to a shortened lifespan in a *Cdk5*α-dependent manner. Means±s.e.m. are presented along the curve. Sample size for control, *Cdk5α*-null, *Cdk5α-*OE and rescue samples were as follows: 485, 518, 472 and 410 male flies, respectively. (D) Loss or overexpression of *Cdk5α* leads to progressive loss of motor function. A partition coefficient (PC) was calculated from the flies’ ability to complete a series of negative geotactic tasks (see Materials and methods). PC is presented as means±s.e.m.; individual replicate PCs are also shown. Five replicates of 20 male flies were analyzed twice for each genotype and time point. Significance of differences is relative to the day 3 control. In all panels, *P<0.05; **P<0.01; ***P<0.001; ****P<0.0001. For the rescue samples, significant differences between rescue and age-matched *Cdk5α*-null samples are indicated as follows: ^####^*P*<0.0001. For full details about sample size, see Table S14. (E) Projected confocal image of adult dorsal notum. Cdk5α-myc, labeled with anti-myc tag (green), and anti-Elav (red) staining. Bright green dots are the tips of sensory dendrites (asterisks) and an axon is indicated. Arrows indicate myc-labeled microchaete cell bodies. (F) Single optical slice of a deconvoluted widefield image stack of thoracic indirect flight muscle. Phalloidin (red) and *Cdk5α*-myc, labeled with anti-myc tag (green), staining. One labeled macrochaete sensory neuron cell body is visible at the lower right (arrow). Scale bar: 50 µm.

The histology of *Cdk5α-*null flies has previously demonstrated age-dependent formation of ‘vacuoles’ in the brain, particularly in the MBs (Trunova and Giniger, 2012). Although these vacuoles are suggestive of neurodegeneration, this assay was not definitive as it did not directly demonstrate neuron loss, as opposed to alternative explanations, such as expansion of inter-neuronal spaces or reduced dendritic arborization. We therefore counted a specific class of MB neurons directly. Flies expressing a nuclear-localized GFP (*UAS-nls-GFP*) under the control of a MB-specific *GAL4* driver [*201Y-GAL4*, which labels gamma neurons and a small subset of alpha and beta neurons (Aso et al., 2009)] were grown to various ages and then dissected. Control flies showed steady numbers of *201Y*-positive neurons through early and middle age, before showing a decline at day (D)45 (mean±s.e.m.: D3 control=763.2±50.3, D30 control=731.3±37.0, D45 control=532.4±72.3 neurons/MB; Fig. 1B). At day 3 and day 10, *Cdk5α-*null flies exhibited a similar number of MB neurons as controls. At day 30, however, *Cdk5α*-null flies showed a sharp decline in neuron number (D30 *Cdk5α*-null=515.0±35.2 neurons/MB, *P*=0.0012; Fig. 1B).

We noted that the average number of MB neurons at day 45 was actually higher in *Cdk5α*-null flies than at day 30 (D45 *Cdk5α*-null=668.3±33.9 neurons/MB), although still less than the number measured at day 3. Based on staining for phosphorylated histone H3, which stains mitotic cells, and EdU, which labels newly synthesized DNA, we found no evidence of neurogenesis (data not shown). Because no new neurons are being born, the simplest hypothesis is that the small fraction of *Cdk5α*-null flies still surviving at day 45 (8.3%) are those that had sustained the least amount of neuron loss with age. Consistent with this, MB neuron number in individual brain hemispheres of the null mutant shows large variance at middle age, including a small population with relatively little cell loss, and experiments suggest that the flies retaining the most motor function at middle age (15 days), as assayed by wall climbing, tend to have more remaining MB neurons than the feeblest flies (data not shown).

In mammals, increased activity of Cdk5 causes neuronal death, as does the loss of function (Patrick et al., 1999). We therefore modestly overexpressed *Cdk5α* and assayed MB neuron survival. We introduced four copies of a transgene bearing the *Cdk5α* genomic locus (Connell-Crowley et al., 2000), which results in an increase of *Cdk5α* mRNA levels by 3.5-fold in head tissue, and 2.2-fold in thorax tissue, as measured by real-time polymerase chain reaction (qPCR) (Table S8C; see below). At 3 and 10 days old, the mean number of *201Y*-positive MB neurons in flies overexpressing (OE) *Cdk5α* appeared lower than controls, albeit not by an amount that was statistically significant (D3 *Cdk5α-*OE=626.8±39.8, D10 *Cdk5α-*OE=576.9±40.2 neurons/MB). By day 30, however, *Cdk5α-*OE flies exhibited a significant decrease in MB neuron number, with a further reduction by day 45 (D30 *Cdk5α-*OE=510.8±27.7, *P*=0.0271; D45 *Cdk5α-*OE=433.9±37.6 neurons/MB, *P*=0.0002). We presume that introducing multiple copies of the genomic *Cdk5α* transgene changes only the level of expression of the gene and not its spatial or temporal pattern. For example, antibody staining of flies bearing four copies of a myc-tagged *Cdk5α* genomic transgene reveals tagged Ckd5α in neurons, but not in muscle, of the dorsal notum (Fig. 1E,F). The expression levels of *Cdk5α* mRNA in all of the experimental conditions were verified by qPCR in an independent sample set; these data are reported in Table S8D (see below). Thus, in the fly, as in mammals, either deletion or overexpression of the gene encoding the Cdk5 activator protein lead to progressive age-dependent neurodegeneration, demonstrated by loss of MB neurons.

### Altered Cdk5α expression induces degeneration-associated phenotypes

We have shown previously that a *Cdk5α*-null mutation causes a variety of degeneration-associated phenotypes, consistent with the neuron loss documented here (Trunova and Giniger, 2012; Connell-Crowley et al., 2007). We now show organismal- and cellular-level degenerative phenotypes in animals with increased *Cdk5α* similar to what was observed in flies lacking *Cdk5α*. In a wild-type (*w+*) genetic background, deletion of *Cdk5α* results in a 31.9% reduction in lifespan relative to control flies (control median survival=47 days; *Cdk5α* null=32 days, *P*<1.0E–15; Fig. 1C). To confirm that the shortened lifespan was a result of Cdk5α levels, we expressed a single copy of the *Cdk5α* genomic transgene in the *Cdk5α*-null flies, and found that one copy of the transgene substantially rescues lifespan, resulting in a median survival nearly equivalent to the control (rescue median survival=44 days; rescue versus control: *P*=0.19; rescue versus null: *P*<1.0E–15). Therefore, the observed decrease in lifespan is caused by reduction of *Cdk5α*. Overexpression of *Cdk5α* had a more severe effect on lifespan than loss of function, as *Cdk5α-*OE flies had a median survival of only 28 days (−40.4% change relative to control, *P*<1.0E–15). Thus, altering the level of *Cdk5α* expression in either direction drastically shortens lifespan.

*Cdk5α* overexpression also caused strong, age-dependent, progressive loss of motor function, as reported previously for *Cdk5α* loss of function (Connell-Crowley et al., 2007). Here, motor function was assayed using an apparatus that gives each fly five sequential opportunities to perform a negative geotaxis task, and reports performance as a partition coefficient (see Materials and methods) (Benzer, 1967; Inagaki et al., 2010). Whereas control flies showed reduced motor function with age, flies lacking or overexpressing *Cdk5α* exhibited a more severe behavioral decline, and with a substantially accelerated time course. Control flies had slight, insignificant decreases in calculated partition coefficient from day 3 through day 30, and then showed a significant decrease at day 45 (Fig. 1D). *Cdk5α-*null flies were nearly identical to controls at day 3, but showed significant decreases at each subsequent time point, whereas the presence of the *Cdk5α* genomic transgene in the null background rescued locomotive ability at each time point (Fig. 1D). Flies overexpressing *Cdk5α* actually started out with severely impaired ability, as their partition coefficient was already significantly lower at day 3, and their motor function remained well below that of controls at all time points, worsening significantly further at day 45. Thus, both gain and loss of *Cdk5α* result in accelerated loss of motor function relative to controls.

**Fig. 2.**
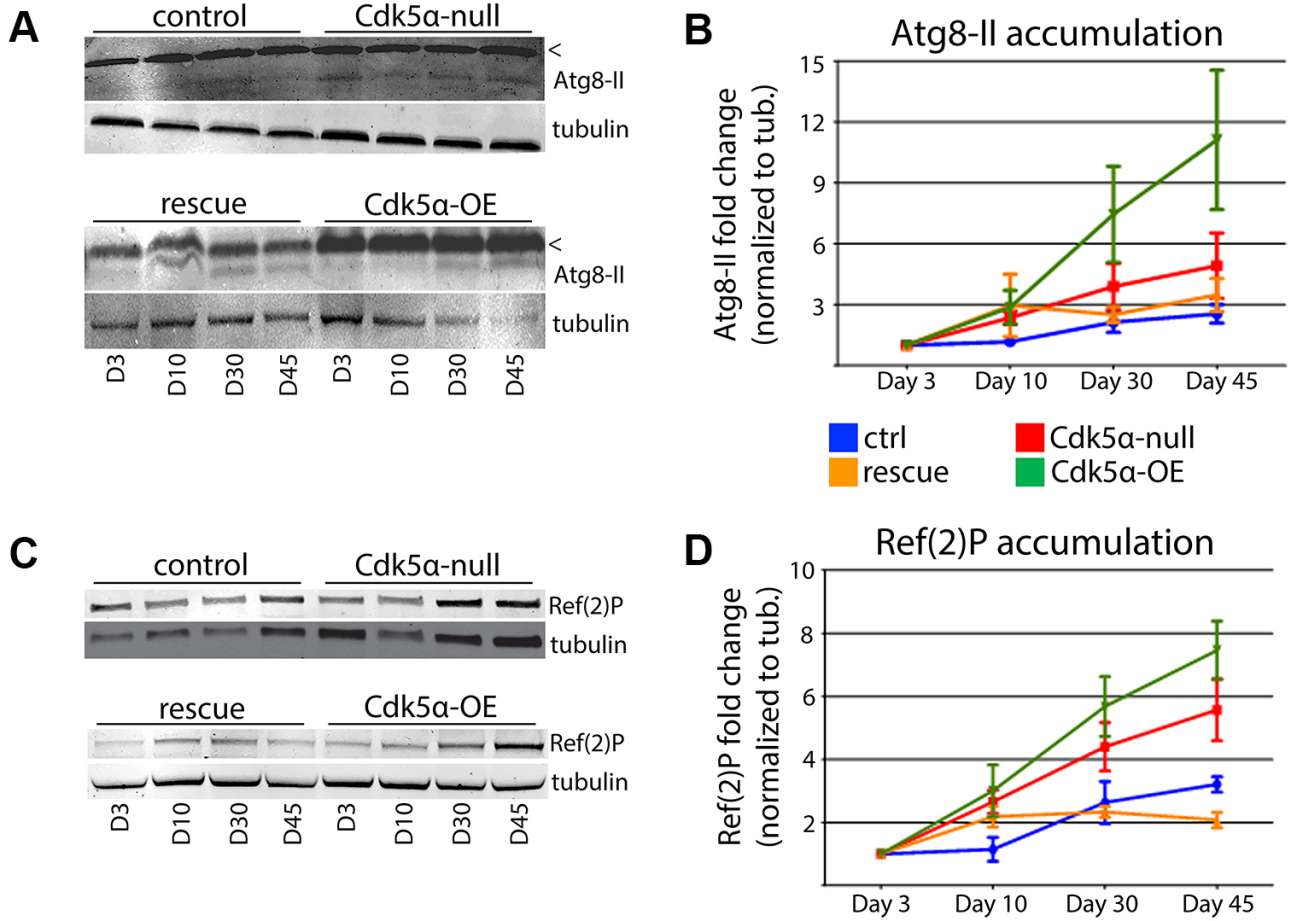
**Accumulation of autophagic markers indicates inhibition of autophagic flux.** Western blot analysis of (A) Atg8-II and (C) Ref(2)P levels in brain homogenate of control, *Cdk5α*-null, *Cdk5α-*OE and rescue flies; tubulin was used as a loading control. (A) Bands marked with ‘<’ refer to the uncleaved Atg8-I. Quantification of (B) Atg8-II and (D) Ref(2)P accumulation based on western blot levels as seen in A and C, respectively; three replicate Atg8-II experiments and six replicate Ref(2)P experiments were analyzed. Values presented are means±s.e.m. For B and D, see text for global comparison of significance of the differences between genotypes across the entire time course for each experiment.

Disrupted autophagy is strongly associated with many forms of degeneration (Hara et al., 2006; Komatsu et al., 2006; Li et al., 2008; Spencer et al., 2009; Winslow et al., 2010). Consistent with this, *Cdk5α* loss of function was previously found to result in an increase of autophagic organelles, as well as an increased sensitivity to starvation, consistent with impaired autophagy (Trunova and Giniger, 2012). As more direct measures, here we assayed Atg8 (LC3 homolog) cleavage and Ref(2)P (p62 homolog) levels as markers of disrupted autophagy. Atg8 is cleaved and lipidated before it binds to autophagosomes and gets degraded (Ichimura et al., 2000); impaired autophagy results in an accumulation of the cleaved form, assayed as an increase of Atg8-II levels. Both loss and overexpression of *Cdk5α* resulted in enhanced accumulation of Atg8-II, relative to control samples. Brain homogenate from control flies revealed a gradual, modest increase in Atg8-II from day 3 through day 45, reaching a maximum 2.5-fold increase at the latest time point (Fig. 2B). *Cdk5α*-null samples, in contrast, showed a 4.9-fold increase in Atg8-II by day 45 [*P*=0.014 for global comparison of the control time course to that of *Cdk5α* nulls; significance assessed by Akaike information criterion (AIC) analysis, applied to non-linear regression of the data] and this increase was partially blocked by the presence of the *Cdk5α* transgene (*P*=0.27 vs control for the complete time course). Samples overexpressing *Cdk5α* showed further exacerbation, as they surpassed the maximum levels observed in controls by day 10, and ultimately reached an 11.1-fold increase in Atg8-II by day 45 (*P*<0.0001 for the complete *Cdk5α-*OE time course relative to control).

*Cdk5α*-null and *Cdk5α-*OE flies also exhibited elevated Ref(2)P levels. Ref(2)P accumulates on autophagosomes and then gets degraded upon fusion of the autophagosome with a lysosome. However, if autophagy is impaired, an increase in Ref(2)P level is observed (Nezis et al., 2008). Whereas control samples showed age-dependent increases in levels of Ref(2)P in brain homogenate, both *Cdk5α*-null and *Cdk5α-*OE brains showed enhanced accumulation at the later time points (Fig. 2D). At day 45, for example, the control samples showed a 3.2-fold increase in Ref(2)P. In contrast, *Cdk5α*-null samples showed a 5.6-fold increase by day 45 (*P*=0.001 relative to control for comparison of the entire time course; AIC test applied to non-linear regression of the data), whereas *Cdk5α-*OE exhibited a 7.5-fold increase in Ref(2)P levels by day 45 (*P*<0.0001 for the complete time course). The *Cdk5α* genomic transgene blocked the accumulation of Ref(2)P in the *Cdk5α*-null background: the rescued samples had only a 2.1-fold increase by day 45, which is comparable to the age-matched control (difference not significant over the time course). Thus, deletion and overexpression of *Cdk5α* both result in significantly elevated levels of Ref(2)P over the lifespan; this is consistent with the Atg8-II accumulation, and supports the hypothesis that autophagy is impaired.

Collectively, these data demonstrate that, as in mammals, either an increase or decrease of *Cdk5α* results in degeneration of susceptible neurons, and induces a variety of characteristic degeneration-associated phenotypes.

### Identification of genes whose expression is altered by Cdk5α and aging

**Fig. 3.**
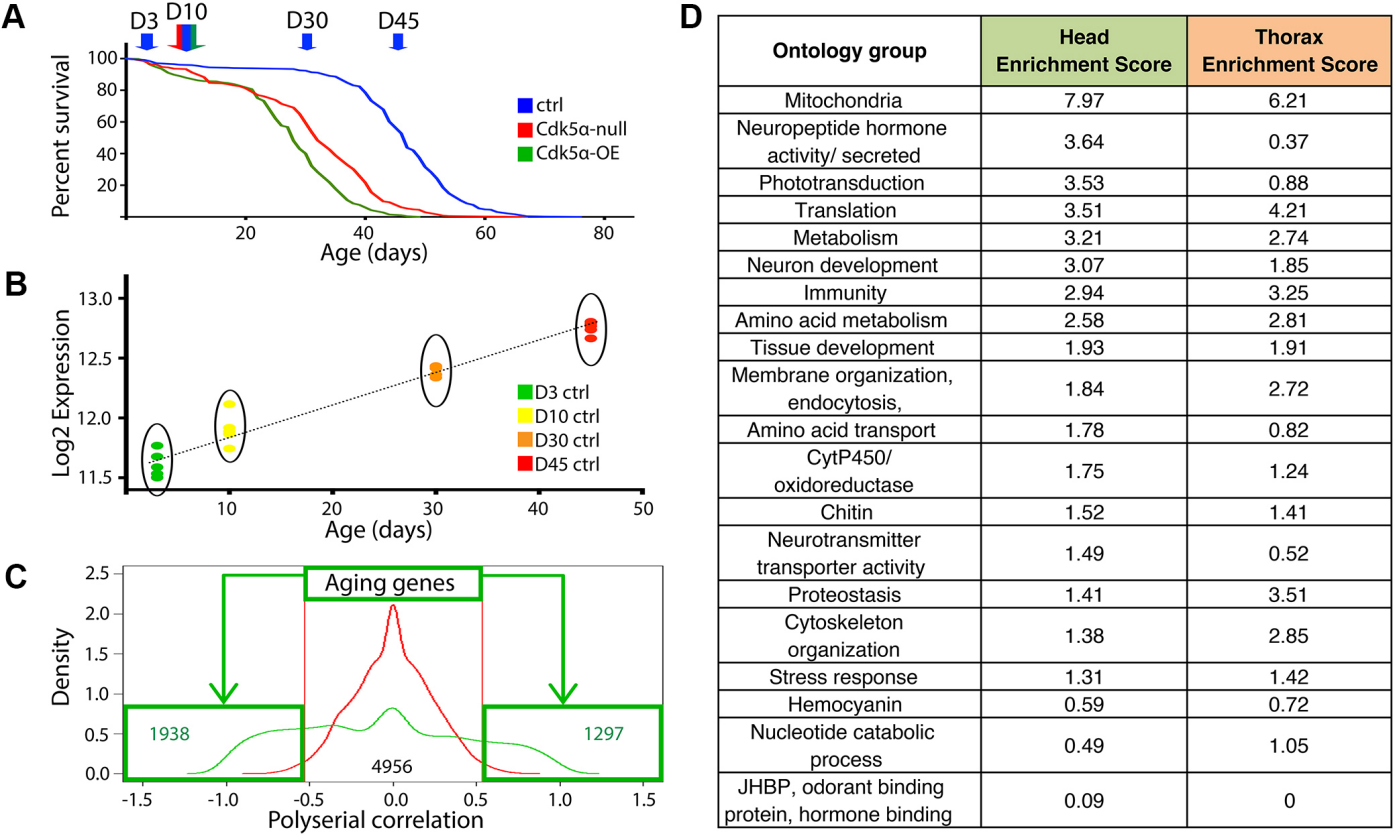
**Identification of aging-related genes and affected biological processes.** (A) Experimental outline schematic. RNA samples were extracted from heads and thoraces of 3-, 10-, 30- and 45-day-old control samples, and from 10-day-old *Cdk5α*-null and *Cdk5α-*OE samples. Five replicates of each sample were collected for microarray analysis. (B) An example of a gene (*Arc1*) showing increased expression with age. Our analysis identified genes with this pattern, or the reverse pattern, as aging-related. (C) Identification of genes positively or negatively correlated with age in head tissue. The green line represents the observed correlation values; the red line represents the correlation estimates when true age is randomized. The randomized set was used to establish significance cutoffs. The vertical red lines indicate correlation values with a corrected *P*-value equal to 0.05 (see Materials and methods). The same procedure was used to define aging-related genes in the thorax (not shown). The full list of aging-related probes is available in Table S1. (D) Gene ontology (GO) analysis of aging-related genes from head and thorax tissue samples. GO analysis was performed on each set of affected probes using DAVID. The resulting annotated clusters were grouped together based on similarity of biological modules; only the highest enrichment score for each ontology group is presented here. Full DAVID results are available in Tables S5,S6. By DAVID's statistical analysis, an enrichment score >1.3 has a *P*-value <0.05.

Several of the phenotypes we observed from altered *Cdk5α* expression resembled a precocious appearance of effects that are seen in natural aging. This led us to wonder whether altering *Cdk5α* might be accelerating the absolute rate of aging. To test this hypothesis, we used genome-wide expression profiling to develop a comprehensive, quantitative and unbiased metric for physiological age. In many systems, it has been found that expression levels of ∼2-30% of genes change in reproducible ways with age (de Magalhães et al., 2009; Girardot et al., 2006; Pletcher et al., 2002). We hypothesized that we could characterize the evolution of the gene expression profile with aging in control flies, genome-wide, and use it as a ‘standard curve’, comparing the profile of a mutant to that of the reference set to infer physiological age (Fig. 3A). We separately isolated RNA from head and thorax of control flies grown to various ages and measured the RNA expression profile using microarrays, with the primarily neural tissue of the head serving as an indicator of neuron-specific changes, and the thorax, which is dominated by non-neural tissue such as muscle, serving as a proxy for systemic changes. We then selected aging-related genes by using polyserial correlation to calculate the association of each gene with aging. Random permutation of expression values with age class was used to establish significance cutoffs, ultimately identifying 3235 and 3809 probes from control head and thorax tissue, respectively, that show consistent changes in expression with age (see Materials and methods; Fig. 3B,C). These probes correspond to 2789 unique genes in the head, and 3245 unique genes in the thorax, with 1657 genes found to be significantly affected by aging in both tissue samples (Table S1).

To validate our set of ‘aging-related’ genes, we first used gene ontology analysis [Database for Annotation, Visualization and Integrated Discovery (DAVID)] to identify biological processes that were over-represented based on their enrichment score (Huang et al., 2009a,b). The set of enriched processes (EASE>1.3) was largely consistent with previous analyses of aging in *Drosophila* (Lai et al., 2007; Pletcher et al., 2002; Zou et al., 2000) and in other organisms (de Magalhães et al., 2009; Golden and Melov, 2004; Lee et al., 1999; Lee et al., 2000), and included mitochondrial function, immunity, proteostasis, and particular aspects of metabolism, among others (Fig. 3D, Tables S5,S6). Similar results were obtained using other gene ontology databases, including Gene Ontology Consortium and Gene Set Enrichment Analysis, for this and all other gene ontology analyses presented below (data not shown).

We next profiled RNA from head and thorax of 10-day-old flies either lacking or overexpressing *Cdk5α*. As above, overexpression was achieved by introducing four copies of a transgene containing the wild-type *Cdk5α* genomic locus, whereas the loss of function was the null mutant. Genes with significantly altered expression were identified by ANOVA under multiple comparison correction condition followed by Tukey-HSD *post hoc* testing. Significance was defined as corrected *P*<0.05 and change in expression level ≥1.5-fold (either increased or decreased) when compared to 10-day-old controls. Loss of *Cdk5α* significantly altered expression of 198 probes (175 genes) in the head and 193 probes (176 genes) in the thorax, whereas *Cdk5α* overexpression significantly affected 328 probes (297 genes) in the head and 405 probes (378 genes) in the thorax (Tables S2,S3).

**Fig. 4.**
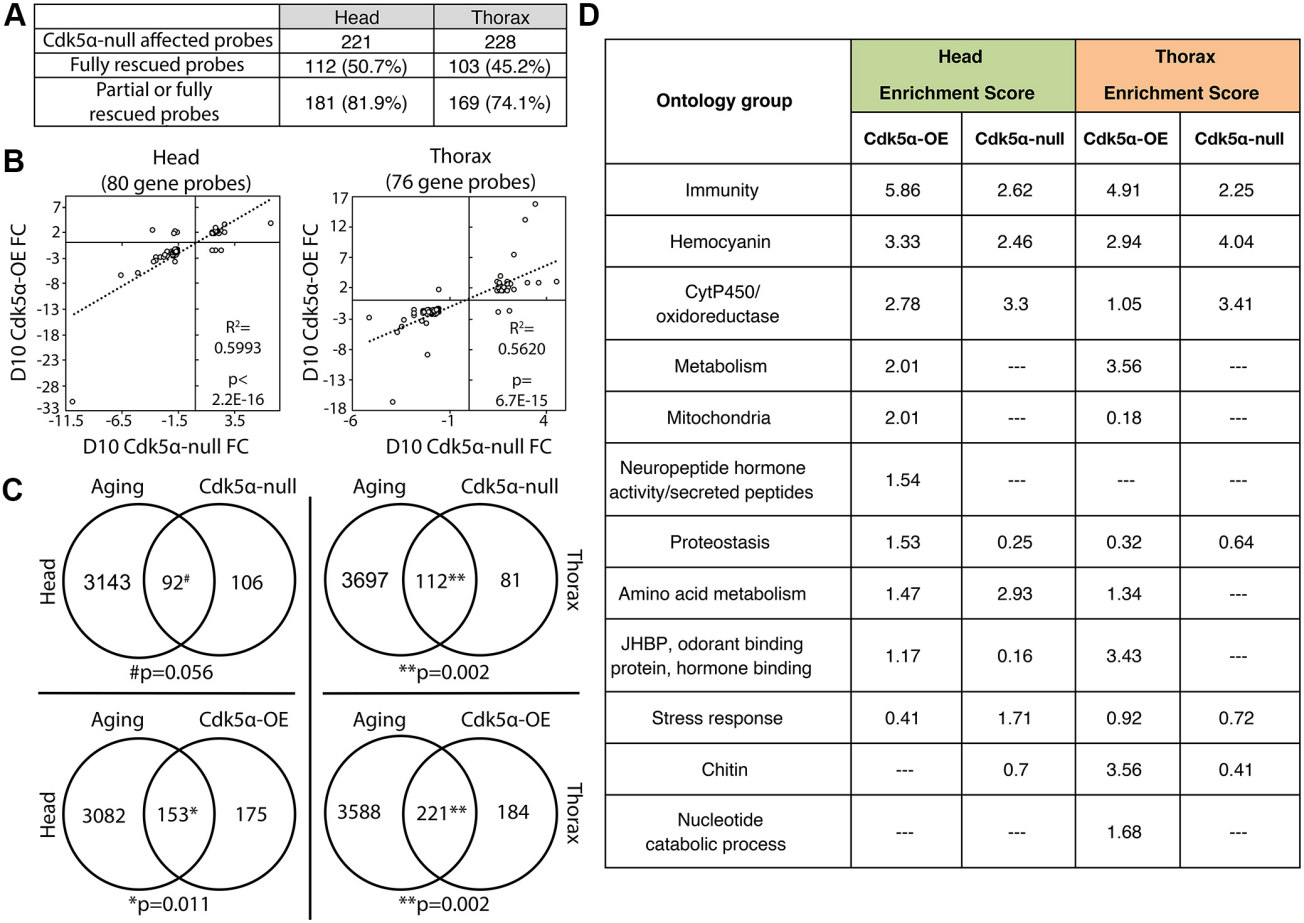
**Identification of genes affected by altered Cdk5α levels, and affected biological processes.** *Cdk5α*-null, *Cdk5α-*OE and rescue samples were analyzed for genes with altered expression relative to age-matched controls. The full list of probes with altered expression is available in Tables S2-S4. (A) The presence of the *Cdk5α* transgene rescues expression changes resulting from loss of *Cdk5α*. Fully rescued probes are those that are significantly altered in the *Cdk5α*-null samples, but are not altered (relative to control) in the rescue samples. Partially rescued probes had their expression levels shifted towards control expression values, but still exhibited >1.5-fold change (FC) relative to controls. Note that the total number of ‘affected probes’ specified in this rescue experiment is slightly different from that reported in the text for the simple gain- and loss-of-function data, since a small number of loci showed differences in calculated statistical significance when the ANOVA included the rescue datasets. (B) Concordance of *Cdk5α*-null and *Cdk5α-*OE intersecting genes in head or thorax tissue. For probes affected by both gain and loss of *Cdk5α*, 91.3% are concordant in head samples, and 96.1% are concordant in thorax samples. (C) Overlap of aging-related probes and probes affected by either loss (upper row) or overexpression (lower row) of *Cdk5α* in head (left column) and thorax (right column) samples. Significance assessed by χ^2^ test, with Yates’ correction. (D) GO analysis of genes affected by gain or loss of *Cdk5α* in head and thorax tissue samples. Enrichment scores are presented as described in Fig. 3; full DAVID results are available in Tables S9-S12.

Three lines of evidence validate the data set and the identification of affected genes. We profiled *Cdk5α*-null flies carrying a single copy of the *Cdk5α* genomic transgene to test whether the transcriptomic effects observed in *Cdk5α*-null flies are indeed *Cdk5α*-specific. The presence of the rescue transgene either fully or partially rescued the expression levels of 81.9% of the *Cdk5α*-null-affected probes in head tissue, and 74.1% of probes in thorax (see Materials and methods, Fig. 4A, Table S4; gene ontology analysis did not identify any consistent, significant differences among the gene ontology groups of genes that were rescued fully versus partially (or not at all) by the transgene (data not shown)]. We next used qPCR to validate 20 genes showing age-dependent or *Cdk5α*-specific changes, in addition to four reference genes, and found that nearly 70% (164/240) of conditions tested were concordant with the array results (Table S8A-C). Last, we observed significant overlap of affected genes between gain and loss of *Cdk5α*, which is consistent with the striking similarity of the degeneration phenotypes observed from either gain or loss of *Cdk5α* expression. Not only was the size of the intersecting set of probes significant (80 probes in head, *P*<2.2E–16; 76 probes in thorax, *P*<2.2E–16), but the probes affected in both *Cdk5α*-null and *Cdk5α-*OE flies were highly concordant (head: *R*^2^=0.60, *P*<2.2E–16; thorax: *R*^2^=0.56, *P*=6.7E–15; Fig. 4B). Among these overlapping probes, the magnitude of expression changes was generally larger in the *Cdk5α-*OE flies, relative to the *Cdk5α*-null samples. This is consistent with the physiological assays, where overexpression of *Cdk5α* typically had more severe phenotypes than did the *Cdk5α*-null mutant.

### The effect of altered expression of Cdk5α mimics aging

#### Gene ontology analysis of Cdk5α-affected genes strongly overlaps that of aging-related genes

**Fig. 5.**
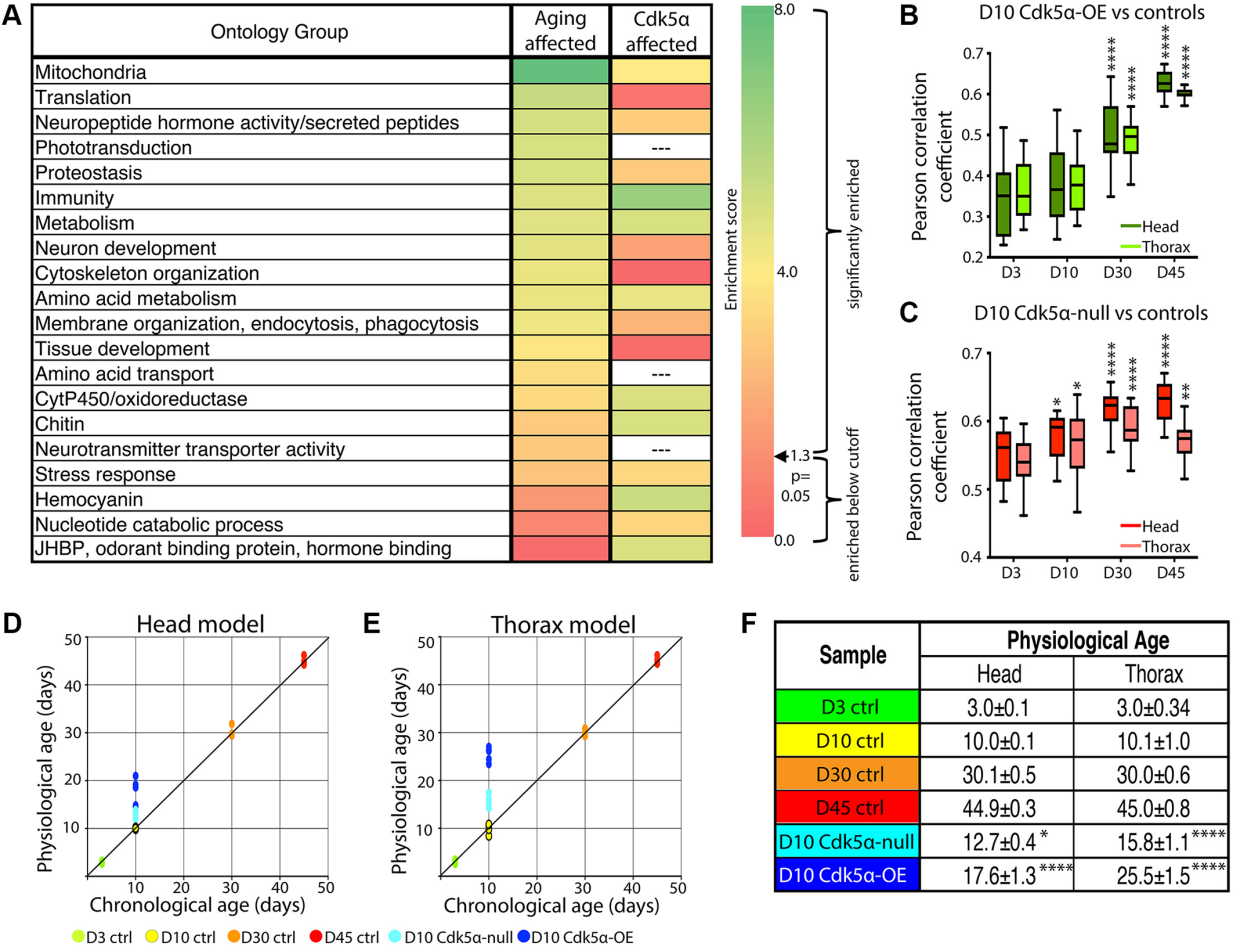
***Cdk5α-*null and *Cdk5α-*OE expression profiles are most similar to older control profiles.** (A) Comparison of significantly enriched GO groups for aging-affected and Cdk5α-affected genes. Heat map is based on DAVID enrichment scores; cells marked with ‘—’ were not enriched. Annotation clusters with an enrichment score >1.3 were significantly enriched; those ≤1.3 were enriched, but not to a degree that reached formal statistical significance. For (B) *Cdk5α-*OE and (C) *Cdk5α*-null samples, the mean expression value of day 10 *Cdk5α*-modified samples was compared to the mean expression value for each of the four control samples, using the intersecting set of *Cdk5α*-affected and aging-affected probes. Box-and-whisker plots show minimum and maximum values. Significant differences between samples are relative to the day 3 correlation values (*P<0.05, **P<0.01, ****P<0.0001). (D-F) Tissue-specific linear models were developed to measure the physiological age of each sample. (D,E) The physiological age is graphed against the chronological age for (D) head and (E) thorax tissue of each sample. (F) The mean physiological age is presented as mean±s.e.m. Significance of differences is relative to the day 10 control samples (*P<0.05; ****P<0.0001).

As a first test of the relationship between the effects of *Cdk5α* and aging, we examined biological processes identified by gene ontology and found a strong overlap between categories enriched by aging and by altered levels of *Cdk5α*. We first noticed a strong overlap between the list of *Cdk5α*-affected genes and the aging-related genes (Fig. 4C). In the *Cdk5α-*OE samples, 153 probes in head and 221 in thorax intersected with the aging set (head: *P*=0.01, thorax: *P*=0.002). In the *Cdk5α*-null samples, 92 probes in head and 112 in thorax overlapped with the aging set (head: *P*=0.056, thorax: *P*=0.002). We then performed gene ontology analysis, which revealed that nine of the top 17 categories enriched in aging-related genes are also among the top categories affected by altered *Cdk5α* expression, including mitochondria, oxidoreductases, metabolism, proteostasis, and immunity (Figs 4D and 5A, Tables S9-12). Some categories that were significantly enriched by the set of aging-related probes were also enriched by the set of *Cdk5α*-affected probes, but at levels further down the enrichment scale: examples include protein translation in the *Cdk5α-*OE samples, and proteostatic processes in the *Cdk5α*-null samples. We also found instances of categories significantly enriched by *Cdk5α*-affected probes but not by aging, as well as categories that differed between head and thorax.

#### Expression profile of affected genes in young *Cdk5α*-null and *Cdk5α*-OE flies resembles the profile in the oldest controls

Comparing the mean expression values of genes affected both by aging and by *Cdk5α* revealed that, for this set of shared genes, the expression profile of young flies with altered *Cdk5α* correlated better with the oldest control profiles than with age-matched profiles. We compared the day 10 *Cdk5α-*null or *Cdk5α-*OE profiles of intersected probes to the control profile at each of the four time points. In the case of *Cdk5α* overexpression, comparing the head profiles from day 10 *Cdk5α-*OE flies with each of the controls yielded average Pearson correlation coefficients of: D3=0.34±0.02, D10=0.38±0.02, D30=0.51±0.02, D45=0.63±0.01 (mean±s.e.m.; Fig. 5B). Similar trends were observed in the thorax, and in both the head and thorax, of *Cdk5α*-null mutants (Fig. 5B,C): in each case, the correlation value of ‘D10 mutant vs D45 control’ was significantly greater than that of ‘D10 mutant vs D3 control’ (*Cdk5α-*OE_Head: *P*=4.6E–10; *Cdk5α-*OE_Thorax: *P*=4.3E–10; *Cdk5α*-null_Head: *P*=4.7E–10; *Cdk5α*-null_Thorax: *P*=0.008). These correlations demonstrate that, for the set of genes that are regulated both by aging and by the expression level of *Cdk5α*, young flies with altered *Cdk5α* have expression profiles more similar to those of older flies. These data are therefore consistent with the hypothesis that aberrant expression of *Cdk5α* does indeed result in an acceleration of at least this portion of the aging process.

Given the neuronal specificity of *Cdk5α* expression, it was unexpected to see such strong effects in thorax tissue: although the thorax includes the thoracic and abdominal ganglia (collectively, the ventral ganglia), its cell mass is dominated by muscle, where *Cdk5α* is not expressed. To rule out the possibility that the observed aging-like changes in thoracic gene expression are driven by RNA from the ventral ganglia, we dissected thoraces, cut them in half, and isolated RNA separately from the ventral half that contains the ganglia, and from the dorsal half that does not. We then performed qPCR on seven test genes. For all seven genes, we found consistent effects of altered *Cdk5α* expression on the RNA profile in the dorsal versus ventral thorax (Table S8E), arguing against the hypothesis that the neural tissue of the ventral thorax is selectively responsible for the ‘aging-mimic’ pattern of overall thoracic gene expression upon modulation of *Cdk5α* level. We note, moreover, that there is precedent for neuron-specific alterations driving systemic changes, and even shifting lifespan of the entire organism (Bahadorani et al., 2010; Kumimoto et al., 2013).

#### *Cdk5α*-null and *Cdk5α*-OE flies are physiologically older than age-matched controls

Experiments above show a strong correlation between the gene expression effects of altering *Cdk5α* level and those of increased age for the specific subset of genes whose expression is regulated, independently, by both of these processes. It remained unknown, however, whether altered levels of Cdk5α mimic only these specific components of aging, or accelerate aging globally. To develop a true gene expression metric for aging, we used machine learning to ascertain ‘aging classifiers’. Using k-nearest neighbor (k-NN) modeling with leave-one-out (LOO) cross validation, we identified individual probes from the control profiles that can be used to estimate the age of an unknown sample based on gene expression levels (see Materials and methods). We then selected the most robust classifiers: those that were identified in every iteration of the kNN modeling (381 and 882 classifiers in the head and thorax, respectively, Table S13). GO analyses performed using a variety of parameter settings verified that the classifiers formed a broadly representative subset of the total group of aging genes described previously. Finally, we used the classifier genes to derive tissue-specific linear models for physiological age based on principal component analysis of the expression data (Fig. 5D,E) and we applied this linear model to the genome-wide gene expression profiles of flies with altered levels of *Cdk5α* to calculate an effective, physiological age for each sample. It is crucial to note that this calculation incorporates the expression levels of all classifier genes for every sample, regardless of whether a given classifier gene is scored statistically as ‘altered’ or ‘not altered’, relative to control, in a particular experimental sample. In the head samples, the linear model measured the physiological age of the 10-day-old *Cdk5α-*OE samples as 17.6±1.3 days (mean±s.e.m.; *P*=7.9E–8, Fig. 5F), nearly twice the chronological age. The *Cdk5α*-null head samples were measured to be 12.7±0.4 days (*P*=0.049), corresponding to a nearly 30% acceleration of aging. Similar results were obtained for the thorax samples (D10 *Cdk5α-*OE: 25.5±0.7 days, *P*=1.8E–14; D10 *Cdk5α*-null: 15.8±0.5 days old, *P*=1.8E–8). These data demonstrate, using a comprehensive, quantitative, genome-wide age metric, that flies overexpressing *Cdk5α* exhibit a physiological age that is dramatically older than its chronological age, both in neural and non-neural tissue, whereas loss of *Cdk5α* results in a subtler, albeit still significant, acceleration of aging.

### Flies with altered *Cdk5α* expression show defects in array-identified biological processes

**Fig. 6.**
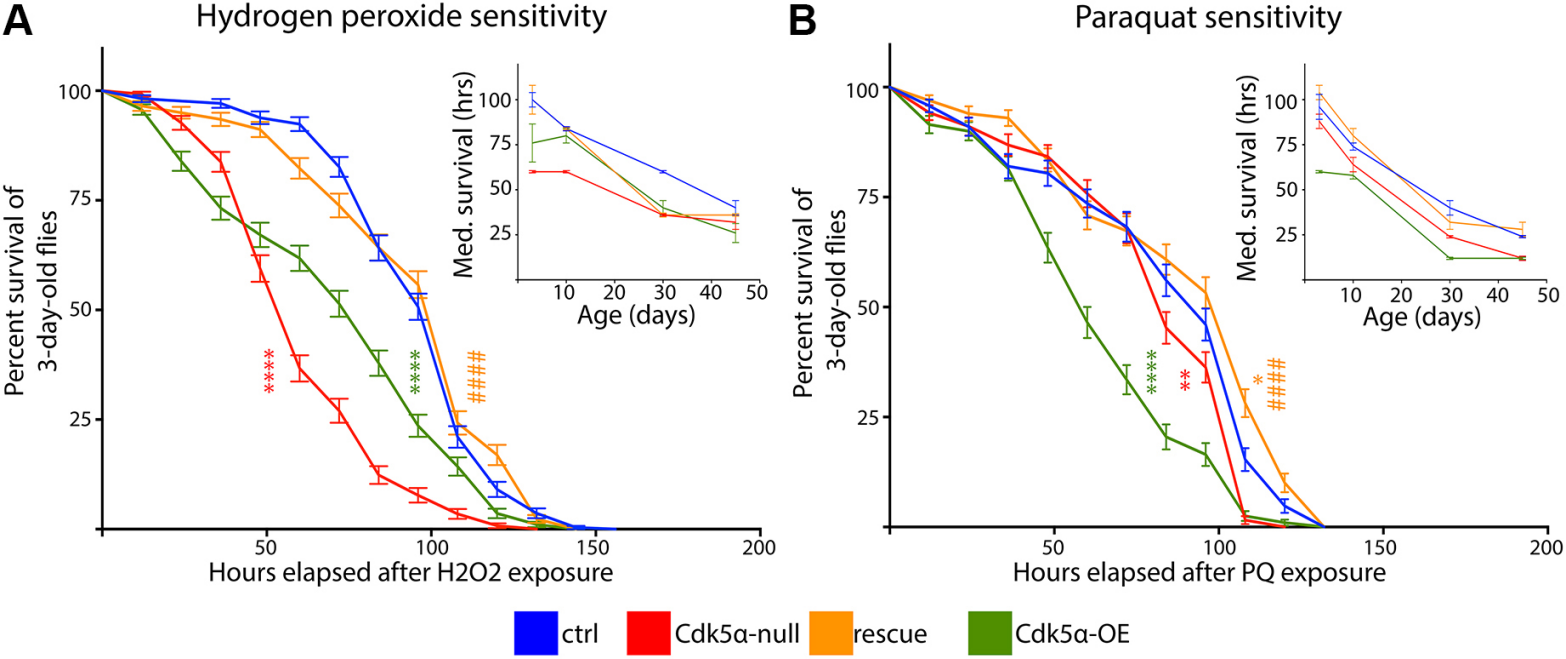
**Altered Cdk5α levels increase sensitivity to oxidative stress.** (A,B) Survival curves of 3-day-old samples exposed to (A) hydrogen peroxide (H_2_O_2_) or (B) paraquat (PQ); error bars represent means±s.e.m. Significant differences of genotypes (vs control) are represented as: *P<0.05, **P<0.01, ****P<0.0001. For the rescue samples, significant differences between rescue and *Cdk5α*-null curves are indicated as follows: ^####^*P*<0.0001. Insets demonstrate the median survival time (in hours) following exposure of 3-, 10-, 30- and 45-day-old flies; error bars represent means±s.e.m. For the experiments depicted in each inset, see text for global comparison of significance of the differences between genotypes across the entire lifespan. Sample sizes are reported in Table S14.

The gene expression data above suggest that there is acceleration of aging with increased or decreased expression of *Cdk5α*. We therefore performed further physiological tests to challenge this interpretation, drawing on processes highlighted by the expression profiling. The gene ontology analysis revealed that genes affected both by aging and by altered *Cdk5α* included a variety of oxidoreductases. We therefore assayed the abilities of flies lacking or overexpressing *Cdk5α* to withstand oxidative stress. Control, *Cdk5α*-null and *Cdk5α-*OE flies were aged to 3, 10, 30 or 45 days and then exposed to hydrogen peroxide (H_2_O_2_) or paraquat (PQ). *Cdk5α*-null and *Cdk5α-*OE flies of each age show enhanced sensitivity to H_2_O_2_-induced oxidative stress, relative to controls. For example, 3-day-old *Cdk5α*-null and *Cdk5α-*OE flies show significantly altered survival curves following exposure (*Cdk5α*-null: *P*<1.0E–15; *Cdk5α-*OE: *P*=6.1E–12; Mantel–Cox log-rank test relative to control; Fig. 6A) and reduced median survival times [control median survival time=100 h, *Cdk5α*-null=60 h (*P*=1.4E–5), *Cdk5α-*OE=76 h (*P*=0.023)]. The detrimental effect in *Cdk5α*-null samples was rescued by the presence of the *Cdk5α* genomic transgene (rescue vs control: *P*=0.39; rescue vs *Cdk5α*-null: *P*≤1.0E–10; rescue median survival time=100 h, rescue vs control: *P*>0.99, rescue vs *Cdk5α*-null: *P*=1.4E–5). Similar trends were observed at all ages, as the median survival time was always lower in *Cdk5α*-null and *Cdk5α-*OE samples than control samples (Fig. 6A, inset), and, for the time course as a whole, both the *Cdk5α*-null and *Cdk5α-*OE flies were more sensitive than controls (*P*<0.0001 and *P*=0.0035, respectively; AIC test applied to non-linear regression of data). PQ treatment also significantly shortened mean survival time of all genotypes following exposure, with the *Cdk5α*-null and *Cdk5α-*OE flies being more susceptible than controls at each individual time point (Fig. 6B, inset), and also globally across the time course (*Cdk5α*-null: *P*=0.0003, *Cdk5α-*OE: *P*<0.0001). Together, these data indicate that both an increase and decrease of *Cdk5α* expression leads to increased susceptibility to various oxidative stresses.

## DISCUSSION

We have shown here that aberrant gain or loss of *Cdk5α* expression accelerates the effective rate of aging in *Drosophila* and induces multiple age-dependent neurodegenerative phenotypes. We exploit the natural modulation of gene expression across the lifespan to define an unbiased, comprehensive and quantitative metric for physiological age. Applying this metric to flies with altered levels of the gene encoding the Cdk5 activator protein, *Cdk5α*, shows that absence of *Cdk5α* increases the rate of aging by >25%, whereas a modest, threefold increase in *Cdk5α* expression causes the aging rate to double. A change of *Cdk5α* levels in either direction, and the attendant acceleration of aging, is associated with adult-onset neurodegeneration, marked by a variety of well-characterized degeneration-associated changes in neuronal physiology, including inhibition of autophagy and sensitivity to oxidative stress, as well as loss of neurons from the central brain, progressive decline in motor function and early death.

Aging is the greatest risk factor for neurodegeneration, but the mechanistic basis for the relationship between degeneration and aging has remained frustratingly enigmatic. One central challenge to clarifying this relationship is the absence of a metric for the physiological age of a subject. Typically, age is defined chronologically, but chronological age is an imprecise, and sometimes misleading, measurement. The rate of aging can be altered by nearly 30% through a combination of genes, environment and happenstance in *Caenorhabditis elegans* (Stroustrup et al., 2016), for example, and lifespan is equally variable in other organisms. Consequently, we sought a more robust quantification of age. Numerous studies document that the expression levels of a significant portion of genes show reproducible changes with age. We therefore assayed multiple points throughout the life of control flies and identified aging-related genes that showed consistent changes in expression with time. We hypothesized that, by comparing a young mutant expression profile to the control profile, we could accurately infer physiological age. This comparison had three potential outcomes: day 10 mutant profiles could have most closely resembled age-matched control profiles, been too disrupted to resemble any particular control profile, or they might have resembled older control profiles.

Our results here display the last of these options: young, essentially presymptomatic, day 10 *Cdk5α-*null and *Cdk5α-*OE samples have an RNA expression profile that strongly resembles that of older control samples. Three separate analyses demonstrate that the phenotype induced by altering the level of expression of *Cdk5α* activity mimics aging. First, we performed gene ontology analysis and found that there is significant overlap in the gene ontology categories enriched among aging genes and those enriched among *Cdk5α*-affected genes. Second, we performed a correlation analysis of the mean expression levels of the particular set of genes that are affected, separately, both during aging and by altered expression of *Cdk5α.* This revealed that, for this set of genes, the samples with an altered level of *Cdk5α* had a pattern of expression more strongly correlated with that of older controls than with the age-matched controls; this increased correlation with older controls was true in both the head and thorax tissue of both *Cdk5α*-null and -OE samples. This shows that altering *Cdk5α* level produces a phenotype that mimics at least this portion of the aging program. Third, we developed a quantitative gene expression metric for age and applied it to RNA from young flies with altered levels of *Cdk5α*. This comprehensive metric allows us to assay the aging process globally, using a genome-wide selection of age-regulated genes. This analysis showed that both head and thorax of the *Cdk5α*-null and *Cdk5α-*OE flies were physiologically ‘older’ than their chronological age, by an amount that is enough to account for a significant portion of the degeneration phenotype. Whether accelerated aging is a universal mechanism of neurodegeneration remains to be seen. A previous study of *Drosophila* neurodegeneration failed to detect expression changes indicative of accelerated aging; in that case, however, mortality was accelerated profoundly by the experimental manipulations, and the compressed timescale may have obscured the ability to detect any age-related effects (Favrin et al., 2013). In contrast, other *Drosophila* models of degeneration show a variety of aging phenotypes, as well as demonstrating aging-related effects on the transcriptome (Kumimoto et al., 2013). Similarly, it has been shown that post-mortem tissue from human AD patients displays a gene expression profile comparable to the profile predicted for non-demented samples at extreme old age, although, in that study, it was not possible to distinguish whether this reflects an early, causal step in disease pathogenesis or a late consequence of terminal disease processes (Podtelezhnikov et al., 2011).

In this study, our goal was to manipulate Cdk5 activity by altering expression of its essential activating subunit, Cdk5α. Mammals have two paralogs of *Cdk5α*, *p35* and *p39*, in addition to one other variant cyclin, Cyclin I, that can stimulate Cdk5 kinase activity (Liu et al., 2017). The *Drosophila* genome sequence, however, reveals only the single *Cdk5α* gene and no *Cyclin I* ortholog. Conversely, we do not detect binding of *Drosophila* Cdk5α protein to classical CDKs, such as Cdk1, 2, 4 or 6 (Connell-Crowley et al., 2000). Moreover, for all phenotypes that have been tested, the effects of deleting *Cdk5* are indistinguishable to those of deleting *Cdk5α* (Connell-Crowley et al., 2007; Kang et al., 2012; Kissler et al., 2009; Nandi et al., 2017). Therefore, although we cannot formally rule out the possibility of non-Cdk5-associated effects of altered *Cdk5α* expression, the simplest hypothesis is that the *Cdk5α* phenotypes demonstrated here arise from altered activity of Cdk5/Cdk5α kinase. Similarly, because overexpression of *Cdk5α* in our study was achieved by introducing *Cdk5α* genomic transgenes at two anonymous locations in the genome, we cannot formally exclude the possibility that some of the effects we observed in the overexpression experiments arose from loss of function of a gene associated with the insertions site(s). We think that this is unlikely, as the putative interrupted gene would have to mimic all of the degeneration phenotypes of *Cdk5α*, both aging-related and aging-independent. Moreover, it is not obvious why heterozygosity for such a hypothetical locus would suppress the phenotype of the *Cdk5α*-null while its homozygosity would mimic the *Cdk5α*-null phenotype. This cannot be excluded, however.

Our data show that increased or decreased expression of *Cdk5α*, presumably with consequent gain or loss of Cdk5/Cdk5α kinase activity, cause similar degenerative phenotypes and neuron loss in flies, just as has been observed in mice and in cultured mammalian neurons. Consistent with this, a change of *Cdk5α* level in either direction accelerates the rate of aging. The expression profiles revealed that a significant number of genes were affected by both loss and overexpression of *Cdk5α*, and these overlapping genes showed very high concordance in directionality. Evidence from mammalian systems suggests that cross-regulatory interactions among proline-directed kinases may be responsible for the similar effects of Cdk5 activation and inactivation. Cdk5 is one member of a group of interacting kinases that have related target-site specificity, including glycogen synthase kinase-3 beta (GSK3β) and mitogen activated protein kinases (MAPK) (Anderton et al., 2001; Hashiguchi et al., 2002; Liu et al., 2002). Consequently, certain key residues of tau, for example, have the same phosphorylation status in both gain and loss of Cdk5 activity, perhaps due to deregulation of GSK3β upon modulation of Cdk5 (Hallows et al., 2003; Hashiguchi et al., 2002; Morfini et al., 2004). If relevant proteins are hyperphosphorylated in similar ways in the context of both increased and decreased *Cdk5α* expression in flies as well, it could explain how both conditions modify the same pathways to produce similar degenerative phenotypes. Alternatively, we cannot formally rule out the possibility that gain and loss of *Cdk5α* lead to degeneration by parallel but distinct mechanisms.

Although an increase and decrease of *Cdk5α* expression give rise to similar phenotypes, hyperactivation consistently produces stronger effects than loss of function, and modulates more gene expression categories in a statistically significant way. It may be that compensation by related kinases is less effective at buffering the effects of kinase hyperactivation than kinase insufficiency. It is also worth noting that we focused our analysis on young, essentially pre-symptomatic flies that had yet to show neurodegeneration, and presumably had yet to exhibit the full effects of altered *Cdk5α* levels on their transcriptome. Moreover, we set relatively restrictive criteria for significance when identifying *Cdk5α*-affected probes, so the set of genes that are affected by altered *Cdk5α* is likely to be rather larger than what we identify. Indeed, when we focus on the 17 gene ontology groups that were significantly enriched for our aging probes, all but three (neurotransmitter transporter activity, amino acid transport and phototransduction) show some level of enrichment among the set of probes altered by aberrant *Cdk5α* expression. These findings suggest that gain and loss of *Cdk5α* likely affect a larger percentage of aging-related genes than demonstrated formally in our analysis.

We do not yet know the mechanism by which Cdk5/Cdk5α kinase modulates the rate of organismal aging. It could be, for example, that Cdk5 kinase directly phosphorylates and regulates some key component of the genetic program that regulates aging. In mice, Cdk5 phosphorylates FOXO3a, a key transcriptional effector of the insulin-like signaling pathway that is thought to control aging in many organisms (Shi et al., 2016). Alternatively, one of the direct, non-aging targets of Cdk5/Cdk5α could potentially have an indirect effect that stimulates some aspect of the aging program (Nandi et al., 2017). Finally, it could be that altering *Cdk5α* expression changes the physiology, or even leads to the degeneration, of insulin-producing cells or of the neurons that regulate them. Additional studies will be essential to discriminate among these hypotheses. For example, it will be interesting to see whether attenuation of aging by activation of FOXO can reverse the age acceleration that we observe upon altering *Cdk5α* expression.

Among the most prominent phenotypes revealed by our expression profiling of altered *Cdk5α* levels are a number that are typically considered early events in the mechanism of neurodegeneration. These include impaired autophagy, which we verified by assaying accumulation of autophagosome-related proteins, and oxidative stress, verified by measuring sensitivity to oxidative challenge with H_2_O_2_ or PQ. However, in our paradigm, much of the disruption of these processes can be accounted for by the observed change in aging rate. We must now consider whether oxidative stress and impaired autophagy, which are usually thought of as degeneration phenotypes, might more accurately be characterized as secondary consequences of altered aging. By extension, we must further consider whether other *Cdk5α*-sensitive pathological processes should really be considered as downstream consequences of the acceleration of aging rather than as causative, early steps in the degeneration cascade *per se.*

It has been argued that neurodegeneration is not simply ‘brain aging’ inasmuch as one can have aging without overt degeneration. Our data do not contradict this view, but rather suggest that aging can promote degeneration, in part, by synergistically enhancing the effects of underlying non-aging insults to neuronal integrity. The *Cdk5α*-null head samples exhibited a 27% increase in aging rate, but showed a severe, localized reduction of MB neurons well before any neurodegeneration was observed in controls. We hypothesize that the increase in physiological age either sensitizes neurons to, or synergizes with, cell-intrinsic defects that we have documented previously as resulting from the absence of Cdk5 activity, such as modulation of the axon initial segment, aberrant organization of actin and ankyrin, or defects in microtubule stability (Smith-Trunova et al., 2015; Trunova et al., 2011). Our findings are thus consistent with the age-dependent hypothesis of AD, which proposes that aging aggravates an initial injury that alters the cellular physiology of neurons and primes them for neurodegeneration (Herrup, 2010), but extend that hypothesis by showing that the underlying mechanism substantially accelerates the time course of aging. In the case of *Cdk5α* overexpression, the aging effect was even more pronounced, and appeared sufficient to account for much of the accelerated MB neuron loss and reduction in median survival, although cell-intrinsic defects likely contribute here as well. Recent experiments with tissue from human AD and PD patients hint that processes similar to those we observe in *Drosophila* may be occurring in human disease, as epigenetic characterization of patient tissue reveals both apparent acceleration of aging (Horvath, 2013; Levine et al., 2015; Podtelezhnikov et al., 2011) and alterations in regulatory marks associated with ankyrin genes that are key to neuronal cytoarchitecture (De Jager et al., 2014; Lunnon et al., 2014). More directed studies of human patient samples will be essential to test this hypothesis.

Using our quantitative metric for physiological age, we observe acceleration of the aging rate upon altering the expression level of Cdk5α and, crucially, we observe this phenotype prior to evidence of neurodegeneration. This discovery seemingly inverts our picture of the causal relationship of aging and neurodegenerative disease, and raises profound questions for our view of neurodegenerative disease. Is it meaningful to use the consequences of age-dependent processes as biomarkers of disease if aging itself is variably affected by the disease mechanism? If tau pathology and associated kinase dysregulation enhance oxidative stress essentially as a downstream consequence of accelerated aging, does it imply that treatments directed at the consequences of oxidative stress would only protect against disease if they reversed aging itself? We suggest that a more complete dissection of the relationship of protein phosphorylation to aging, and to neurodegenerative disease, is likely to enhance our ability to advance treatments for aging-associated disorders, such as ALS, PD and AD.

## MATERIALS AND METHODS

### Fly stocks

Control flies were from an Oregon Red wild-type background (*w+*). *Cdk5α* loss-of-function conditions [previously termed *p35^20C^* and *Df(p35)^C2^*] have been described previously (Connell-Crowley et al., 2000, 2007; Smith-Trunova et al., 2015; Trunova et al., 2011; Trunova and Giniger, 2012). The *Cdk5α*-null alleles were crossed into the control background (*w+; Cdk5α^20C^*). Overexpression of *Cdk5*α was achieved by adding four copies of a transgene encoding the *Cdk5α* genomic locus (Connell-Crowley et al., 2000) in the same background (*w^+^; P[w^+^,Tn Cdk5α]^R244^/P[w+,Tn Cdk5α]^R244^; P[w+,Tn Cdk5α]^R157^/P[w+,Tn Cdk5α]^R157^*). For assaying the spatial pattern of *Cdk5α* in the overexpression context, the same strategy was used, but employing homozygosity for two insertions of an equivalent genomic transgene bearing a myc-tagged version of *Cdk5α* (Connell-Crowley et al., 2000). The ‘rescue’ line was generated by putting a single copy of the transgene into the *Cdk5α*-null background (*w^+^; Cdk5α^20C^Cdk5α^20C^; P[w+,Tn Cdk5α]^R157^/+*). For MB cell counting, the original *UAS-nls-GFP* fly stock was obtained from the Bloomington *Drosophila* Stock Center. The γ-neuron-specific *GAL4* driver *201Y-GAL4* was used to express *UAS-nls-GFP* in control (*w^+^; 201Y-Gal4/+; UAS-nls-GFP/+*), *Cdk5α-null* (*w^+^; DfC2,201Y-GAL4/Cdk5α^20C^; UAS-nls-GFP/+*) or *Cdk5α-OE* (*w^+^; 201Y-GAL4,P[w+,Tn Cdk5α]^R244^/P[w^+^,Tn Cdk5α]^R244^; UAS-nls-GFP*, *P[w^+^,Tn Cdk5α]^R157^/P[w^+^,Tn Cdk5α]^R157^*) backgrounds.

### MB neuron counting

Control, *Cdk5α*-null and *Cdk5α-*OE male flies carrying single copies of *201Y-GAL4* and *UAS-nls-GFP* were aged to 3, 10, 30 or 45 days. Whole brains were microdissected and fixed in 4% paraformaldehyde, then mounted on slides with VectaShield (Vector Laboratories, Burlingame, CA). Microscopy was performed on a Zeiss NLO510 confocal microscope or with the Zeiss Yokogawa spinning disk (SD) system mounted on an inverted Zeiss Axio Observer Z1 microscope; images were acquired using a 40× oil objective. *Z*-stacks were collected from individual brain hemispheres, and were analyzed using Bitplane's IMARIS (version 7.5.2) and its ‘Add spots’ function to semi-automatically count the labeled neurons; false positives were manually removed and false-negative nuclei were manually added. Control experiments revealed no significant difference in counts from *Z*-stacks acquired on the NLO510 versus the SD. Furthermore, the expression of *201Y-GAL4* does not diminish with age in control or *Cdk5α*-null brains, indicating that the decrease in labeled cells reflects cell loss and not loss of marker expression (data not shown).

### Tissue staining, antibodies and general microscopy

Fluorescence microscopy was performed either on a Zeiss 880 confocal or a Zeiss AxioImager widefield microscope. Tissue fixation was by standard methods (Trunova et al., 2011). Antibodies used were as follows: anti-Elav [mAb 7E8A10, 1:10 dilution; Developmental Studies Hybridoma Bank (DSHB), Iowa City, IA], rabbit anti-myc tag (1:500; Sigma-Aldrich, St Louis, MO). Alexa-Fluor secondary antibodies (1:500) and phalloidin (1:200) were from Molecular Probes/Life Technologies, Grand Island, NY.

### Lifespan assay

To assay lifespan, 10-14 male and 10 female newly-eclosed flies were aliquoted into a vial containing standard cornmeal-molasses food and a strip of tegocept paper. Flies were maintained at 25°C with a 12 h:12 h light:dark cycle. Flies were transferred to fresh vials every 3 days; dead male flies were counted at each transfer until all flies had died.

### Motor function assay

Motor function was assayed using a modified version of the phototaxis assay originally developed by Benzer (Benzer, 1967); our version of the apparatus had six tubes on the lower frame and five tubes on the upper frame. For the assay, the flies were slightly anesthetized under CO_2_, divided into replicates and transferred to fresh vials. After waiting for 3 h to let the anesthesia wear off, flies were transferred to the first tube of the lower frame, and then given 3 min to acclimate to the apparatus. Once acclimated, the apparatus was gently tapped down on the table five times; this served to pool the flies at the bottom of the tube, as well as to agitate the flies, which induces negative geotaxis. The top frame is then slid to the left, such that the flies can climb from their current tube at the bottom into the first tube on the top. After 20 s, the upper frame is slid to the right. The apparatus is gently tapped down on the table again, depositing all of the flies that climbed up into the top tube down into the second tube on the bottom frame. This process is then repeated four more times, ultimately resulting in the flies being distributed throughout the six bottom tubes based on their climbing ability.

A partition coefficient (PC) was calculated using a weighted average as described by the formula below (Inagaki et al., 2010):

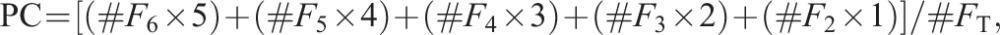


where #*F_n_* is the number of flies in tube ‘*n*’, and #*F*_T_ is the total number of flies. Percent change was calculated relative to the day 3 control {100–[100×(PC_Sample_/PC_D3ctrl_)]}. Percent rescue was calculated as 100×[(PC_Rescue_ – PC_Cdk5α-null_)/(PC_Control_ – PC_Cdk5α-null_)] at each time point. The PC is superior to simple counting of flies that reach a fiducial mark, as in the standard wall-climbing assay (Connell-Crowley et al., 2007), for three reasons. First, by giving each fly five chances at the task it intrinsically smooths the data for what is otherwise a quantitatively noisy assay; second, by reporting the entire distribution of climbing abilities it gives additional quantitative data; and third, by providing physical separation of flies with different motor capabilities it facilitates further analyses.

### Western analysis

Twenty male flies of each genotype were collected at the defined age (3, 10, 30 and 45 days), then flash frozen with liquid nitrogen and stored at −80°C. Heads were separated from the rest of the body and homogenized in lysis buffer (2% SDS, 150 mM NaCl, 50 mM Tris, pH 7.5) containing protease inhibitors (Thermo Scientific, Pierce, Rockford, IL). Supernatant protein concentrations were determined using the Coomassie Protein assay reagent (Thermo Scientific). Protein samples (25 μg) were resolved on a 4-12% Bis-Tris plus gel (Invitrogen/Thermo Fisher Scientific, Carlsbad, CA) and transferred onto iBlot2 NC membranes using the iBlot 2 system (Invitrogen/Thermo Fisher Scientific). Membranes were cut to separate bands based on molecular mass, and then probed with primary antibodies overnight at 4°C. Primary antibodies used included anti-Ref(2)P (1:1000; gift from Sheng Zhang, University of Texas, TX) (Rui et al., 2015), anti-dATG8 (1:500; gift from Sara Cherry, University of Pennsylvania, PA) (Shelly et al., 2009) and anti-β-Tubulin (catalog #E7, 1:500, DSHB, Iowa City, IA). Infrared fluorescence IRDye secondary antibodies, including goat anti-rabbit IgG (H+L) 800CW, goat anti-rabbit (680RD) and/or goat anti-mouse (H+L), were applied for 20 min at room temperature (1:5000, LI-COR Biosciences, Lincoln, NE) followed by washing with PBS. Visualization and quantification was carried out with the LI-COR Odyssey scanner and software (LI-COR), with tubulin serving as a loading control.

### Microarray and mRNA expression analysis

For microarray analysis, five biological replicates for each genotype and time point were collected. Each replicate consisted of 140 male flies, aged as described for the lifespan assay; following aging, collected totals ranged from 90 to 140 flies. We did not use any formal *a priori* statistical methods to predetermine sample sizes; rather, sample sizes were selected based on recommendations from the microarray literature to account for variability. Replicates consisted of pooled tissue samples to provide sufficient material for microarray and qPCR analysis. Upon collection, flies were transferred to a 1.5 ml Eppendorf tube and flash frozen in liquid nitrogen, then stored at −80°C. All time points were collected and stored prior to processing. Eppendorf tubes containing frozen flies were transferred into liquid nitrogen to further chill them, then vortexed to separate the heads, wings and legs from the rest of the bodies. Keeping the samples cold on dry ice, the heads were manually transferred to a fresh 1.5 ml Eppendorf tube. Thoraces were separated from the abdomen using a surgical blade and then transferred to their own tube. Total RNA was extracted for both head and thorax samples using TRIzol (Invitrogen), then processed and labeled according to the manufacturer's guidelines for use with the DroGene 1.0 ST GeneChip (Affymetrix, GeneChip Whole Transcript Sense Target Labeling). The Scanner 3000 (Affymetrix) was used in conjunction with GeneChip Operation Software (Affymetrix) to generate one .CEL file per hybridized cRNA. Two separate batch runs were required due to logistical reasons, with a common technical replicate sample included in both batches. The first batch run included control samples from four time points (day 3, 10, 30 and 45) plus 10-day-old *Cdk5α*-null and *Cdk5α-*OE samples; the second batch run included day 10 control and day 10 rescue samples. The Expression Console (Affymetrix) was used to summarize the data contained across all .CEL files and generate 16,322 robust multiarray average (RMA) normalized gene probe expression values. Subsequent analysis of these values was then performed separately for head and thorax. Specifically, quality of expression was challenged and assured via Tukey box plot, covariance-based PCA scatter plot, and correlation-based heat map using functions supported in ‘R’ (www.cran.r-project.org, data not shown). Following this initial inspection, a single day 3 control head sample presented itself as a cohort-level outlier; this sample was removed and not included in any downstream analysis. To remove batch effects between the two runs, baseline subtraction was performed using expression for a common technical replicate present across batches followed by use of quantile normalization to correct for differences in spread. To remove noise-biased expression values, we used lowess modeling to look for a relationship between mean gene expression and the corresponding coefficient of variation (CV). Lowess fits were then over-plotted to identify the common low-end expression value where the relationship between mean expression (signal) and CV (noise) deviated from linearity (mean expression value=7.5). Expression values less than this value were set to equal 7.5, whereas gene probes not having at least one sample greater than 7.5 were discarded as non-informative. Annotations for genes observed to have differential expression and/or modeled were obtained from NetAffx (Affymetrix) and FlyBase (www.flybase.org). Full probe lists are available in Tables S1-S4.

Polyserial correlation (library=polycor) was used to generate estimates of how expression observed per gene linearly relates to age in control samples. These estimates were in turn compared to those obtained when true age is randomized, with estimates greater than or less than two standard deviations of the mean of random generated estimates being considered as significant (*P*<0.05); genes passing these conditions were deemed to be aging-related. To identify Cdk5α-related genes, ANOVA testing was applied using sample class as the factor under Benjamini–Hochberg false discovery rate multiple comparison condition. Gene probes observed to have a corrected *P*-value <0.05 by this test were further *post hoc* challenged via Tukey-HSD test. Gene probes observed to have a *post hoc P*-value <0.05 and an absolute difference of means >1.5-fold for a class comparison were considered to have differential expression between the two classes. Chi-square with Yates’ continuity correction was used to determine the significance of the overlap between gene lists. The linear relationship between overlapping probes was tested with Pearson's correlation analysis.

To test similarities in the expression profiles, the mean expression value of the set of genes affected by both aging and altered Cdk5α was calculated. Each individual day 10 *Cdk5α*-null or *Cdk5α-*OE sample was compared to each day 3, 10, 30 and 45 control sample, generating a set of Pearson correlation coefficients. Comparing each of five *Cdk5α*-null replicates to four day 3 control head and five of every other control sample yielded 95 total comparisons for head tissue, and 100 comparisons for thorax tissue. The same number of comparisons was used for *Cdk5α-*OE. Significance was determined using one-way ANOVA with Tukey's multiple testing correction (MTC).

To identify aging classifiers, leave-one-out (LOO) testing was employed using the same methods described above, but on gene expression not discarded for day 3, 10, 30 and 45 control samples only. For each LOO round, gene probes deemed to have differential expression for at least one class comparison were used to construct a k-nearest neighbor (k-NN) model and predict the class of the left out sample (Dudoit et al., 2002). Gene probes selected 100% of the time over all LOO rounds were deemed control aging classifiers. These genes were then used to construct a principal component seeded AIC-optimized linear model using expression for day 3, 10, 30 and 45 control samples only. This model was used to predict the physiological age of each biological replicate (four day 3 control head samples, and five of every other sample and time point). Statistical differences in the predictions produced by the linear model were determined by performing one-way ANOVA with Tukey's MTC on the predictions themselves.

To test whether expression changes observed in the *Cdk5α*-null samples were indeed a result of a decreased *Cdk5α* expression level, a transgene carrying the *Cdk5α* genomic locus was added to the *Cdk5α*-null background. Genes were considered fully rescued if they showed ≥1.5-fold change (based on microarray data) relative to day 10 controls in the *Cdk5α*-null samples, but not the rescue samples. Partially rescued genes were those with expression values that trended towards the control values but still exhibited ≥1.5-fold change relative to day 10 controls.

### Gene ontology analysis

Gene ontology analysis was performed using annotated probes identified as aging-related or Cdk5α-related (see Statistical analysis section below), using version 6.7 of DAVID (http://david.abcc.ncifcrf.gov; Huang et al., 2009a,b). The background was set to ‘Drosophila_2 Array’, and then ‘Functional Annotation Clustering’ with medium classification stringency was used to identify groups that were significantly enriched. An enrichment score greater than 1.3, correlating to a non-log scale *P*-value less than 0.05, was considered statistically significant. The resulting annotated clusters were grouped together based on similarity of biological modules; only the highest enrichment score for each ontology group is presented here. Full DAVID results are available in Tables S5,S6 and S9-S12.

### qPCR validation

Excess RNA from the microarray samples was converted into cDNA using the Applied Biosystems High Capacity cDNA Reverse Transcription kit; three biological replicates were run in triplicate for every gene probe. qPCR reactions were prepared using the Affymetrix VeriQuest Probe qPCR Master Mix with specific TaqMan gene primers (Table S7); reactions were carried out on the Bio-Rad iQ5 Multicolor Real-time PCR Detection System. Threshold cycle numbers were determined automatically by the Bio-Rad software.

The set of probes included four reference genes (*eIF-1a*, *Rap2L*, *Rpl32* and *Sdha*), which were used to compute a geometric mean for normalization. Fold changes were determined using ΔΔCt (Livak and Schmittgen, 2001), relative to day 3 controls (for aging-specific changes) or day 10 controls (for mutant-specific changes).

### Oxidative stress sensitivity assay

Sensitivity to oxidative stress was assayed by survival following challenge with paraquat (PQ; 15 mM), which catalyzes the production of superoxide and induces oxidative stress (Farrington et al., 1973), or hydrogen peroxide (H_2_O_2_; 5%), which induces oxidative stress through the generation of reactive hydroxyl radicals (Halliwell and Gutteridge, 1984). Male flies were mated for 3 days, and then females were removed. Males were then aged to 3, 10, 30 or 45 days, starved for 4 h on filter paper damp with water, and then transferred to vials containing filter paper moistened with 5% sucrose solution and PQ or H_2_O_2_. A separate set of flies received only 5% sucrose as control (data not shown). Survival was scored every 12 h, and median survival time calculated. Three biological replicates were analyzed for each genotype and time point. Each replicate consisted of 2-5 vials of 7-20 males each (D3 control=189, D10 control=175, D30 control=196, D45 control=256, D3 *Cdk5α*-null=190, D10 *Cdk5α*-null=150, D30 *Cdk5α*-null=199, D45 *Cdk5α*-null=259, D3 rescue=199, D10 rescue=147, D30 rescue=209, D45 rescue=240, D3 *Cdk5α-*OE=200, D10 *Cdk5α-*OE=179, D30 *Cdk5α-*OE=223, D45 *Cdk5α-*OE=239 total flies); individual replicate sizes are presented in Table S14.

### Statistical analysis

All data were analyzed with GraphPad Prism 7.0b and R. Sample sizes for all experiments are outlined in Table S14. Differences between groups were assessed by either one-way or two-way ANOVA with *post hoc* Tukey multiple comparison testing, as described. Statistical differences in survival curves (lifespan and oxidative stress response) were measured by log rank (Mantel–Cox). All statistical tests were two-tailed, and statistical significance was considered at *P*<0.05. Statistical significance of differences between genotypes in time course experiments was determined by non-linear regression, using the Akaike information criterion (AIC), as implemented in GraphPad PRISM 7.0b. Experimental groups were determined based on genotype, so no randomization was used; data collection and analysis was not performed blind to the conditions of the experiments. Sample sizes were not predetermined using statistical methods. Data distribution was assumed to be normal with equal variance, although this was not formally tested.

## Supplementary Material

Supplementary information
