# Supplementary material for: Altered expression of the Cdk5 activator-like protein, Cdk5α, causes neurodegeneration, in part by accelerating the rate of aging
Source: Dis Model Mech. 2018 Mar 1;11(3):dmm031161. doi: 10.1242/dmm.031161 (PMC5897722; doi:10.1242/dmm.031161)
Supplement: Supplementary information [file dmm-11-031161-s1.pdf]

Supplemental Tables

Supplemental Table 1. List of gene probes altered by age from head and thorax tissue.
Gene probes identified as aging-related in either head or thorax tissue are provided; the Affymetrix gene probe number and corresponding Flybase gene assignment and gene symbol are included.

Click here to Download Table S1

Supplemental Table 2. List of gene probes altered in *Cdk5α*-null head and thorax tissue.
Gene probes in either head or thorax from *Cdk5α*-null background exhibiting ≥1.5-fold change relative to age-matched controls are provided; the Affymetrix gene probe number and corresponding Flybase gene assignment and gene symbol are included.

Click here to Download Table S2

Supplemental Table 3. List of gene probes altered in *Cdk5α*-OE head and thorax tissue.
Gene probes in either head or thorax from *Cdk5α*-OE background exhibiting ≥1.5-fold change relative to age-matched controls are provided; the Affymetrix gene probe number and corresponding Flybase gene assignment and gene symbol are included.

Click here to Download Table S3

Supplemental Table 4. List of gene probes altered in D10 rescue head and thorax tissue.
Gene probes in either head or thorax from rescue background exhibiting ≥1.5 fold change relative to age-matched controls are provided; the Affymetrix gene probe number and corresponding Flybase gene assignment and gene symbol are included.

Click here to Download Table S4

Supplemental Table 5. Gene ontology results from list of genes altered by aging in head tissue.
Full gene ontology results from DAVID analysis on the list of head aging-related probes. Annotation clusters that were deemed significant following DAVID's criteria (enrichment score >1.3) are highlighted.

Click here to Download Table S5

Disease Models & Mechanisms • Supplementary information

Supplemental Table 6. Gene ontology results from list of genes altered by aging in thorax tissue.
Full gene ontology results from DAVID analysis on the list of thorax aging-related probes. Annotation clusters that were deemed significant following DAVID's criteria (enrichment score >1.3) are highlighted.

Click here to Download Table S6

Supplemental Table 7. List of qPCR primers used for validation of microarray data.
The gene symbol, gene name, probe number, and catalog number are provided for each qPCR primer used for validation.

Click here to Download Table S7

Supplemental Table 8. qPCR validation of microarray results.
(A) Summary of agreement between microarray and qPCR results. Fold change values from microarray and qPCR analysis were "in agreement" if both values were positive or both were negative. (B) Aging-related trends observed from control samples. Fold change was calculated relative to the Day 3 control sample. (C) Changes resulting from altered Cdk5 activity. Fold change was calculated relative to the Day 10 control sample. For the sample labels of both (B) and (C), "H" refers to head samples and "T" refers to thorax samples. "---" indicates samples that were not detectable above background by qPCR. (D) *Cdk5α* levels were verified by qPCR of RNA from an independent set of 10-day old head samples of each genotype. "---" indicates that *Cdk5α* was undetectable in the *Cdk5α*-null samples; *Cdk5α* levels were normalized to Rpl32. (E) Comparison of expression fold changes, relative to control samples, in ventral and dorsal thorax samples from *Cdk5α*-null flies.

Click here to Download Table S8

Supplemental Table 9. Gene ontology results from list of genes altered in *Cdk5α*-null head tissue.
Full gene ontology results from DAVID analysis on the list of probes affected by deletion of Cdk5α in head tissue. Annotation clusters that were deemed significant following DAVID's criteria (enrichment score >1.3) are highlighted.

Click here to Download Table S9

Disease Models & Mechanisms • Supplementary information

Supplemental Table 10. Gene ontology results from list of genes altered in *Cdk5α*-null thorax tissue.
Full gene ontology results from DAVID analysis on the list of probes affected by deletion of Cdk5α in thorax tissue. Annotation clusters that were deemed significant following DAVID's criteria (enrichment score >1.3) are highlighted.

Click here to Download Table S10

Supplemental Table 11. Gene ontology results from list of genes altered in *Cdk5α*-OE head tissue.
Full gene ontology results from DAVID analysis on the list of probes affected by overexpression of Cdk5α in head tissue. Annotation clusters that were deemed significant following DAVID's criteria (enrichment score >1.3) are highlighted.

Click here to Download Table S11

Supplemental Table 12. Gene ontology results from list of genes altered in *Cdk5α*-OE thorax tissue.
Full gene ontology results from DAVID analysis on the list of probes affected by overexpression of Cdk5α in thorax tissue. Annotation clusters that were deemed significant following DAVID's criteria (enrichment score >1.3) are highlighted.

Click here to Download Table S12

Supplemental Table 13. List of aging classifiers from head and thorax tissue.
Full list of robust aging classifiers identified in every round of leave-one-out testing in either head or thorax tissue; the Affymetrix gene probe number and corresponding Flybase gene assignment and gene symbol are included.

Click here to Download Table S13

Supplemental Table 14. Sample sizes for all experiments.
The sample size of each genotype at each time point for all experiments is outlined. For experiments utilizing survival curves (Fig 1C, Fig 6A-B), both the total number of flies and the sample size of each replicate is included.

Click here to Download Table S14
